# An evaluation of online Edcraft gamified learning (EGL) to understand motivation and intention of recycling among youth

**DOI:** 10.1038/s41598-022-15709-2

**Published:** 2022-12-12

**Authors:** Kin Meng Cheng, Ah Choo Koo, Junita Shariza Binti Mohd Nasir, Shen Yuong Wong

**Affiliations:** 1 Faculty of Creative Multimedia, Multimedia University, Cyberjaya, Malaysia; 2 School of Electrical Engineering and Artificial Intelligence, Xiamen University Malaysia, Selangor, Malaysia

**Keywords:** Environmental impact, Psychology and behaviour, Sustainability

## Abstract

Recycling is a process carried out by various organizations and individuals to enhance the environment’s long-term sustainability. Some youth think that recycling is a monotonous action as it may seem inconvenient, less aware of the environmental issues and more time-consuming than they think and rather go for video games. Therefore, this study investigates the relationship between motivation and recycling intention in gamified learning among youth. To address the research aim, this study uses gamification as a motivational driver for a game-like learning experience to improve recycling intentions among youth. Self-determination theory (SDT) and the theory of Planned Behavior (TPB) will be this study's main motivational and behavioral theories. (n = 124) high schools and college students were invited to take part in an online gamified recycling activity, Edcraft Gamified Learning (EGL), consisting of two levels of gamified unused plastic-crafting recycling activities. After the activity, the participants will answer a post-event questionnaire and the data collected were analyzed. The result shows that controlled motivation (CM) and autonomous motivation (AM) positively influenced youth attitudes and social norms. Besides, attitude is the only psychosocial determinant that positively influences the recycling intention of the youth. Gamification only moderates positively between attitude and recycling intention. This study has clearly shown the effectiveness of gamified learning activity towards recycling intention directly and as a component that moderates the relationship between attitude and recycling intention, which shows a favorable evaluation towards recycling intention with gamified learning involved. Moreover, the findings showed that not all relationships are positive in a gamified learning environment, and it gives a good view on the weakness and strengths with the guideline of SDT and TPB.

## Introduction

Sustainable environment for the habitat. In Malaysia, the increase in the amount of waste has led to a higher recycling rate. According to Compendium of environment statistics, in Malaysia, the waste generated from 2018 to 2019 is from 3098.7 tonnes to 3108.9 tonnes, an increment of 0.33%. However, the recycling rate has increased from 24.6% (2018) to 28.1% in 2019. It was expected that the waste would increase to 30,000 tonnes daily by 2020^1^. However, by May 2019, 38,000 tonnes of waste generated daily from different sources like industrial waste, household waste and services waste^2^, which exceeded the expected amount of waste generated.

Among all the waste in the environment, plastic is one of the most difficult recycled substances, an estimated of 8300 million metric tons (Mt) of plastic has been produced, but only 9% is recycled, and the rest are landfilled or incinerated^3^.

Recycling potential has been studied, Malaysia’s recycling rate of 28.1% is still far from the recycling rate in some of the higher recycling performance countries with the latest available data from the year 2014–2019, such as Singapore at 59%, South Korea at 60% and Hong Kong at 36.5%^4,5^.

Waste generated is often landfill, legally or illegally dumped into rivers and seas, without the awareness about the consequences that will potentially negatively impact the ecosystem's environmental health. This is where the learning of recycling occurs to get more awareness among the people, especially the youth, in recycling. Youth (aged 15 to 24 years old) account for 17% of the world’s population, or 1.2 billion people, with the majority (87%) living in developing countries, such as Korea, Singapore and Malaysia. As the next generation of people and national leaders who will lead the society, provide innovative and groundbreaking solutions, it is crucial to educate them on the consequences of climate change causing by unethical disposal and waste management^6^. As video game elements show effectiveness in engaging a classroom, Gamification is a form of engaging learning in a fun environment and has been used in various educational settings^7,8^. The behaviorist learning theory on motivation shares many characteristics with gamification, including challenges, responsive feedback, interaction, development, and upgrades^9^.Therefore, Self-determination Theory (SDT) is one of the guiding theories to learn the motivational aspects of this study.

Sustainability has been widely studied in the past several decades, and youth are the group with a special focus on several Sustainable Development Goals (SDG), education (SDG 4) and Climate Action (SDG 13)^10,11^. The theory of planned behavior (TPB) also will be analyzed as the youths’ volunteerism towards the external environment. By assessing the regularity of people’s psychological and behavioral intentions, this theory assists managers, psychologists, or researchers to plan constructive measures and achieve organizational objectives^12–15^. A study by Strydom^16^ discussed the barriers of household recycling, majority of the respondents have claimed that the four major reasons for not recycling are lack of time, following by the awareness, insufficient space, and convenience of recycling. It is believed that awareness of the environmental threat caused by unmanageable waste are needed, and this will change the thought of the people to spend more time to recycle even within a smaller or less space. Recycling behavior is said to consider the convenience and ease of use and the advantage given to a person to act, internally or externally stimulated motivation^17^.

The use of gamification may influence the recycling intention of people to a certain degree, and gamification is a moderator in this research to identify whether it impacts the recycling intention with the guidance of self-determination theory and theory of planned behavior. There are only a few research studies on gamified learning and recycling intention. Besides, recycling also has been known for its lack of participation from the public due to the less interest or less exposure about the importance of environmental sustainability^18^.

This research uses SDT and TPB as the guided theory to close the gap between youth’s motivation and recycling intention on gamified learning. A study from Chaba et al.^19^ showed the use of autonomous and control motivation in SDT to predict the socio-cognitive variables (i.e., attitude, subjective norm, perceived behavioral control, and intention to gain muscle mass) from TPB. It shows a strong relationship between the SDT and TPB. Therefore, this study developed two research questions (RQ), in which RQ 1 is how the controlled, autonomous motivation, and psychosocial determinants influence recycling intention among Malaysian youth through gamified learning; and RQ 2 is with and without gamified learning, how the recycling intention differs.

## Motivation in gamified learning and recycling intention

This section will discuss two significant parts of the literature that guides the study on the motivational difference of recycling intention with and without gamified learning in Sect. 2.1, then the two guided theories on SDT and the TPB. Due to the TPB's static explanatory nature, it is difficult to comprehend the shown impacts of behaviour on cognitions and future behaviour. Therefore, the conceptualization of the framework also considers SDT, which has a higher prediction over the TPB^20^. Following with Sect. 2.2, the hypothesis to understand the motivation, recycling intention. Then the gamified learning that could be the moderator to enhance youth’s intention on recycling will answer RQ 1 and RQ 2.

The motivation of gamification started as early as the 1980s, and it was used to describe the implementation of games and non-game context^21^. Gamification aims to increase people’s engagement and promote motivation for positive behaviors^22,23^. In the education domain, gamification is widely used, and through the use of game elements (i.e., leaderboards, levels, points), it is believed to be able to engage learning and providing “fun” and “enjoyment” to learners in different subjects^24,25^. With the increasing popularity of online learning due to the COVID-19 pandemic, students are to study from home, and there is a need to apply gamified learning to engage students in learning effectively.

Gamification is believed to be the concept that fit learning and game elements to achieve learning goals if planned strategically in applying rewards, levels, points and challenges into conventional learning. Implementing gamification strategies in an online learning environment like gamified intervention or gamified activities increases intrinsic motivation, learning performances, encourages learners to engage in challenging and creative activities, and help broaden the spectrum of potential participants and application scenarios, relatively a change of behavior that involves retention^26–28^. Moreover, gamified learning covers various advantages from other learning strategies like peer-learning strategies that focus on aiding the peer group in learning and active learning strategies that focus on student engagement in activities stimulating higher-order thinking, problem-solving, and critical analysis feedback^29,30^.

Therefore, Self-determination theory, was used in a wide range of gamification research and created a two-way relationship between motives and learning. Findings also showed that the gamification mechanism in learning shaped excitement in participatory experience and encouraged learners to act voluntarily^31^.

Learning can be on any subject, but particularly this study is on recycling, which is also an action to achieve environmental sustainability. Recycling behavior is widely studied by academia and practitioner, and the theory of planned behavior has been the influential model over the years.

A study from Juliana et al.^32^ and Gadiraju^33^ about waste management on crafting unused items has been carried out to increase public awareness of recycling, exposing the youth’s idea about recycling possibilities. TPB was used to analyze the outcome, shows that recycling activities without motivational affordance like game elements will only improve the knowledge, attitude, and view towards recycling. Therefore, this has proven that motivation is needed to enhance the recycling intention, and the following part will further evaluate the literature of the motivation and recycling intention on gamified learning.

### Self determination theory (SDT)

Self-determination theory (SDT) has been widely used in human motivation and personality in social contexts that differentiate motivation^34^. SDT is termed the basic needs theory as it gives satisfaction in learning and as a drive to continue to progress with a task or a mission^35^. Therefore, SDT has been the form of foundation to gamification as this theory also shows it utilitiaristic (reusability) and hedonic (pleasure) variables that include intrinsic and extrinsic motivation on SDT continuum and applied in few studies in gamification and psychological effectiveness to achieve learning outcomes^36–40^.

**Figure 1:**
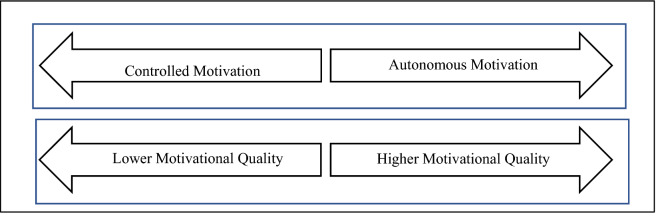
Adapted from SDT continuum: controlled motivation and autonomous motivation in motivational quality^43^.

According to the adapted continuum of SDT in Fig. 1 shown below, the controlled and autonomous motivation continuum can gauge the lower or higher motivational quality^41^. Autonomous motivation is fully within the person’s willingness, choice and coalition in an activity. In contrast, controlled motivation is reflected as to oblige to work with the task^41,42^.

#### Autonomous motivation (AM)

Derived from SDT, autonomous motivation has a higher motivation effect quality than controlled motivation and does not require external pressure^44^. Autonomous motivation refers to behaviors based on a person’s will because of enthusiasm, fun, or challenge. It feels like their own identity has been recognized, and the actions are meaningful and personally important. Autonomous motivation, especially identified regulation and interest as an integral part of intrinsic motivation, corresponds conceptually to the intrinsic value of the Expectancy by Value Model^45,46^.

Autonomous motivation leads to own purpose (recycle intention) when people are following their interests and carry out tasks and activities that can draw their attention. A person may develop a sense of own purpose, that will lead them to a more functionality and efficiency in completing task^44^. Luqman et al.^47^ mentioned that autonomous motivation and TPB can assist in alleviate negative behavior and influenced the intention of an individual and develop better behavior^48^. It is believed that autonomous motivation and TPB are both useful to create a better attitude towards recycling.

#### Controlled motivation (CM)

Derived from SDT, controlled motivation is referring to the behaviors of learners feeling when compelling to participate in internal or external psychological pressures event, which drives them to involve due to the avoidance of unwanted outcomes, behaviors that learners engage in to improve their self-esteem or escape feelings of remorse and shame or behaviors that they embrace to obtain contingent incentives or avoid negative consequences^49^.

When they are controlled motivated, participants are pressured externally, monetary reward or to avoid punishment or internally, from the satisfaction, completion of a certain task. As such, youth are not volunteered to join the activity as they are supposed to be. Consequently, when controlled motivated, students are expected not as fully functional as they are as compared to autonomous motivated as they are internally volunteered^34^. However, controlled motivation is believed an individual tend to follow peers and influenced by them to change attitude, which potentially given them the belief in the act of recycling. controlled motivation and TPB will be suitable to influence the recycling intention of the youth.

### Theory of planned behavior (TPB)

The TPB has been widely used in the study of recycling intention^50^, this theory is developed to explain a variety of human behavior in many areas of research fields, including business, marketing, information technology and education^51^. In the educational context, students who tested with TPB positively impacted their participation in MOOC (Massive Open Online Course)^52^. Besides, teachers have also been tested by TPB and found the improvement in the willingness to use technology in teaching^53^. Fishbein and Ajzen^54^ proposed that if the target behavior is measured under the three psychosocial determinants, Attitude, Perceived behavior control, and the subjective norm is necessary to understand and predict the impact of individual moral consciousness on behavioral intention^13^.

Ajzen and Fishbein^55^ mentioned that attitude is the first determinant of behavioral intention in the TPB model. Behavioral beliefs usually resulted from favourible and unfavourible attitude, a positive attitude refers to the degree of a person’s positive favourible on a certain interest. On the other hand, if someone has a negative or unfavourable mindset on certain interest, their action intention would be lower^56^. Yzer^57^ differs attitude into two types, attitudes on non-behavioral objects (e.g., kids, shops and motivation) and attitudes towards performing a specific behavior (e.g., exercise, jogging and singing). This study will mainly focus on attitudes towards behavioral intention (Recycling intention). Studies show that one’s attitude positively affects one’s behavior^58^. However, studies also show that the students have positive attitudes, but they do not put their behavioral intentions into action towards environmental behavior^59^. Therefore, gamified activity takes into consideration to elicit their favouritism from a fun and engaging learning in environmental behavior and potentially put the behavioral intentions into practice.

Subjective norm refers to the social pressure that people experience when deciding whether to do anything. The views of family members, friends, societies, and government departments can easily influence an individual’s decision to engage in a particular behavior. Several research studies have shown a positive relationship between subjective norm and behavioral intention^60^. Here, online gamified activity might have a positive outcome if there is a positive influence among friends within the activity.

Perceived behavioral control (PBC) is another determinant of behavioral intention in the theory of planned behavior, and it applies to people’s perceptions of how easy or difficult it is to execute the behavior of interest^13^.Several factors correlate with the capacity and convenience of recycling use and influence youths’ behavioral intention. For example, the experience of plastic bottle crafting, video game and perception of the importance of recycling would affect youths’ behavioral intention in recycling. Those who hold a strong PBC will practice recycling around their surroundings^61^. When a youth has a high experience and has a better understanding of recycling, they would be more likely to apply to recycle in their life if they have a high experience in crafting and have a greater understanding of video game mechanism the gamified learning activity is more challenging. As a result, it is thought that perceived behavior control has a beneficial impact on the behavioral intention in recycling.

#### Recycling intention

Attitude, social norm, and perceived behavioral control affect behavioral intention. The behavioral intention in this study is recycling intention, which is the probability or a purpose to perform a behavior or action like recycling based on people’s expectancy^62^, the expectation to perform a task such as homework, household chores and recycling. The behavioral intention is the degree to which someone will attempt a specific task, as well as the amount of effort they plan to put forward, also referred to as behavioral intention, the participation of the task is from they own willingness^55,63^. A deep desire to participate in a behavior will eventually contribute to that behavior^64^. Residents’ perspectives were examined in this report. TPB has focused on the determinants that will lead to the behavior, but there is much to explain from a person’s motivation performing a task. Therefore, to tackle motivation, the following section will go further into the context of motivation. This study believes that the behavioral intention is not only from attitude, perceived behavioral control, and social norm but also to gain more insightful relationship between motivation areas, such as SDT.

### Gamification

Deterding et al.^65^ described that gamification is the application of game-like design elements to non-game contexts. It has been a term in academia for around 10 years and was coined in 2002 by Nick Pelling^66^. It can be used in education, industries, and government programs. However, because of its broad application and application to several systems, its principles must be revised regularly^67^. Therefore, in this study, several game elements were used: leaderboard, points, badges, levels, clear goals, and rewards. These elements mentioned by Hamari et al.^68^, are the motivational affordances in gamification and are generally used in empirical gamification studies. Besides, it serves as an environment for fun learning^65^ that leads to the fostering of students’ motivation.

In this study, gamification is applied in learning and used to explore the relationship between gamification in learning (gamified learning), autonomous motivation and controlled motivation with TPB. Based on SDT, need-supporting gamification will intensify learners’ autonomous motivation. However, most game elements used in a gamified environment favor controlled motivation^69^. The inclusion of gamification in an educational and learning context should foster motivation without adding feelings of controlled motivation^70^. However, in learning, controlled motivation can be transfer learning that the motivation does not initiate by the individual, that makes gamification takes its place to initiate the controlled motivation of recycling, as well as autonomous motivation, that initiated from their internal value and drive in recycling intention. Therefore, the following hypothesis is formulated.

Five main hypotheses have been formulated to address this study’s two RQs, as shown in Table 1. RQ1 has led to the hypotheses H1 and H2, the relationship to controlled motivation and autonomous motivation towards the psychosocial determinants of recycling intention. Lastly, on H3, the relationship between psychosocial determinants and recycling intention is discussed.

**Table 1:** Research hypotheses.

**H1: Autonomous motivation positively improves psychosocial determinants on youth’s recycling intention:**
H1a—Autonomous motivation (AM) has a positive association with attitude of recycling,
H1b—Autonomous motivation (AM) has a positive association with perceived behavioral control of recycling
H1c—Autonomous motivation (AM) has a positive association with subjective norm of recycling
**H2: Controlled motivation positively improves psychosocial determinants on youth’s recycling intention:**
H2a—Controlled motivation (CM) has a positive association with attitude of recycling,
H2b—Controlled motivation (CM) has a positive association with perceived behavioral control of recycling
H2c—Controlled motivation (CM) has a positive association with subjective norm of recycling
**H3: Psychosocial determinants positively improve youth’s recycling intention:**
H3a- Attitude has a positive association with recycling intention
H3b- Perceived behavioral Control has a positive association with recycling intention
H3c –Subjective Norm has a positive association with recycling intention
**H4: Gamification positively moderates between psychosocial determinants and youth’s recycling intention:**
H4a –Gamification moderates between attitude and recycling intention
H4b—Gamification moderates between perceived behavioral control and recycling intention
H4c—Gamification moderates between subjective norm and recycling intention
**H5: Gamification positively improve youth’s recycling intention**

In RQ 2, gamification, which is gamified learning, leads to the generation of H4, where gamification acts as a positive relationship of psychosocial determinants and recycling intention and H5, the direct relationship between gamification and recycling intention.

**Figure 2:**
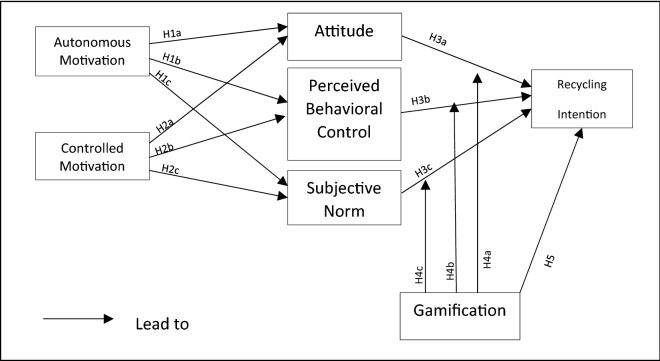
Conceptual model of motivation and recycling intention on gamified learning.

The research conceptual model is proposed in Fig. 2, to investigate the relationship of EGL recycling activity of the youth towards the recycling intention with the hypotheses formulated above.

### Methodological procedures

124 youths were invited from secondary and tertiary schools from Selangor, Malaysia, and 100 youths have participated in the online gamified recycling activity assisted with Google workspace services, namely, Edcraft Gamified Learning (EGL) activity. Prior to the invitation involving youths, a poster was sent to the corporate communications unit to verify and approve licensing and legal issues. The research target samples were well confirmed and approved by the dean of the Faculty of Creative Multimedia, Multimedia University Malaysia (MMU) that the researcher affiliated. Besides, the methodological procedures, rules and activities had been shared with the dean of the university and the research participants before the EGL activity started to confirm all the methods were carried out in accordance with relevant guidelines and regulations as approved by the Multimedia University’s secretariat of Research Ethics Committee, ethical approval (EA) number, EA2642021 and reference number, TTO/REC/EA/264/2021.

EGL will apply game elements of points, level, challenges and leaderboard along with the recycling craft activity, where the participant will be the player of EGL to complete challenges from two levels, points achieved from the two levels will determine their position in the leaderboard and the top 15 highest points participants will be rewarded.

The host of this study manages the entire session of the activity. They will be the person to lead the online gamified activity and link the activity with participants and the jury team. The jury consists of a pool of evaluators comprising university lecturers and researchers with Multimedia and art backgrounds. Their role is to use the evaluation form provided by the host to evaluate the recycling craft of the participants, and the participants are the youths from higher secondary school and college students with age range between 16 to 28 years old, as confirmed by the ministry of youth in Malaysia in 2019 Youth Societies and Youth Development Act (Amendment) 2019 (Act 668).

### The EGL activity flow

Each participant will experience an online, two days, two levels, each level per day plastic-bottle crafts EGL activity. The EGL started with a briefing session for the participants to understand the activity's rules and regulations. Then, each individual was given informed consent (online Google form) to confirm their voluntary participation before the activity. The information is given, including a statement explaining the study, purposes, expected length of time for participation, predictable risks, possible outcomes, and subject participation, is entirely voluntary. Once they are clear with the information and have filled up the form, they will watch a video regarding recycling awareness and a sample video of the level 1 crafting tutorial, a used plastic bottle made plastic vase. After watching the two tutorial videos, participants were required to create a video out of their crafting process, and they were given the freedom to craft within 10 h. Once finished, they will upload their video to Google drive’s dedicated folder. The Jury team evaluated the participants’ crafts based on their creativity, innovation, video presentation and completeness to fulfil the rubrics tailored based on this study, and the result of the first leaderboard will be shown before starting level 2.

The next day, they will go through the same process in level 2, but with the higher difficulty of recycling craft, a plastic-bottle made face shield, they will upload their crafting process video for level 2 craft, and their work will be evaluated once more. The total average of the two levels points will determine their position on the leaderboard, and every participant can see their own and peers’ results from Google Sheet. By the end of the activity, All participants will earn a certificate of appreciation and be positioned on top 15 on the leaderboard will be rewarded with prizes based on the points they gained. All participants were to fill a post-activity survey questionnaire which comprised questions as shown in Table 2.

**Table 2:** Validity and Reliability of Constructs.

Measurement items	Loadings	AVE	CR
***Autonomous motivation***			
I recycle because I feel that I want to take responsibility for the earth	0.81	0.921	0.598
I feel like I want to recycle	0.797		
I recycle because I personally believe it is the best thing for earth’s health	0.673		
I recycle because I have carefully thought about it and believe it is very important for many aspects of my life	0.772		
***Controlled motivation***			
I recycle because it is an important choice I really want to make	0.801		
I recycle because it is consistent with my life goals	0.695		
I recycle because it is very important for being as recycling conscious as possible	0.85		
I would feel guilty or ashamed of myself if I am not recycle	0.748	0.831	0.419
Others would be upset with me if I not recycle	0.691		
I recycle because I would feel bad about myself if I not recycle	0.792		
I recycle because I feel pressure from others if they recycle	0.613		
I recycle because I feel the pressure from my peer if they practice recycle	0.62		
***Gamification***			
I think Information Technology and Multimedia can assist in recycling	0.74	0.872	0.434
I think Information Technology and Multimedia will assist in recycling intention in daily life	0.739		
I prefer to have challenges or Quest in a recycling activity	0.638		
I would expect to have a mentor/teacher to complete the challenges when recycling	0.65		
I think completion of an activity from level 1 to 2 can create sense of accomplishment	0.762		
I think leaderboard is an important element to increase participation among peers in recycling activity	0.617		
I think leaderboard impacted in gamified classroom	0.676		
***Attitude***			
I feel like I am saving the world if I practice recycling	0.884	0.907	0.662
I feel better about myself afterwards if practice recycling	0.806		
I feel positive about myself if I practice recycling	0.853		
I realized recycling is enjoyable if I practice recycling	0.833		
***Perceived behavioral control***			
The recycling space and atmosphere around my neighborhood influences my decision to do recycling	0.839	0.893	0.736
The facilities at my local recycling space influence my decision to do recycling	0.836		
Safety at my local recycling space influences my decision to do recycling	0.897		
***Subjective norm***			
My friends think I should recycling	0.925	0.894	0.739
My family think I should recycling	0.874		
My peers do recycling more, so I ought to do it too	0.773		
***Intention***			
I am willing to recycle now	0.847	0.813	0.457
I intend to recycle recyclable plastic items	0.843		
I plan to recycle more, especially unused plastic	0.862		
I will often recycle plastic consumption in the future	0.857		
I will recycle the used items such as plastic	0.896		

### Research instrument

Creswell (2010) suggested that a cross-sectional survey is an appropriate method to “examine attitudes, beliefs, opinions and practices. The questionnaire consists of 42 questions and is categorized into seven parts, “autonomous motivation”, “Controlled motivation”, “Gamification”, “attitude”, “subjective norm”, “perceived behavioral control”, and “intention”. These constructs were measured using a 5-point Likert scale ranging from 1 (strongly disagree) to 5 (strongly agree). 5-point Likert scale has widely been used by health science and behavioral research that involve Theory of Planned Behavior for validity and reliability. The sources from which the questionnaire was produced were summarized in Table 1 and adapted from the SDT survey by Williams^71^.

## Results

The survey questionnaire will follow a systematic approach of Path Modeling analysis using SmartPLS. SmartPLS is a form of Structural Equation Modeling that concurrently estimates and tests causal among several dependent and independent variables^72^. After filtration of the survey response, 40 out of 100 participants did not complete the EGL activity, questionnaire, or dropouts, leaving only 60 participants who had completed the Edcraft Gamified Learning (EGL) activity post-survey questionnaire, it fulfils the sample-to-variables ratio of 5:1 and 15:1 or 20:1 as suggested by Matthews et al.^73^. The total independent variables are 6 (controlled motivation, autonomous motivation, attitude, perceived behavioral control, subjective norm and gamification). Therefore, 30 respondents have to acquire as the minimum requirement for the analysis.

To begin, SPSS version 27.0 was used to perform descriptive statistics and reliability analysis on the data collected from the post-survey questionnaire and evaluate the sample and the constructs’ internal consistency. Then, using SmartPLS 3.0 software, as Partial Least Squares (PLS) is a technique used widely for estimating path coefficients in structural models and used in several research studies on the study of behavioral intentions even under the condition of non-normality and small to medium sample sizes to model latent constructs^74^.

The PLS path model assessment using the suggested two-stage analytical procedures for SEM, outer model assessment (observation level) and inner model assessment (theoretical level)^75^. Outer model assessment tested on convergent validity, then the measurement model was tested to measure the validity and reliability and then examined the structural model. On the other hand, inner model assessment consisting of variance of endogenous constructs, effect sizes and predictive relevance^76^.

A bootstrapping method (500 re-samples) was used to measure the significance of the path coefficients and loadings^74^. Structural equation modeling (SEM) requires data not to breach the assumption of normality, therefore, a check on normality of the data is needed, a general guideline for skewness is that if the number is greater than + 1 or lower than –1, this shows a substantially skewed distribution^77^.The skewness ranging from -1.588 to 0.837, therefore it is substantially skewed. However, the Kurtosis statistics ranging from -0.987 to 4.469. Kline^78^ mentioned if some values of Kurtosis exceeded 3, the data was considered as violating normality. Hence, a partial least square (PLS) based SEM was used for this study.

## Measurement model assessment

### Convergent validity

As above mentioned, the researcher will first examine the outer loadings, composite reliability, average variance extracted (AVE). The analysis started with measuring each item of the construct including Autonomous Motivation (AM), Controlled Motivation (CM), Attitude (ATT), Perceived behavioral Control (PBC), Social Norm (SN) and Recycling intention (INT) with standardized factor loadings^74^. As a standard rule of thumb by Hair et al.^74^, and recommendation by Fornell and Larcker^79^, the value of factor loadings preferable to be ≥ 0.70. However, ≥ 0.50 are accepted for exploratory research. As shown in Table 1, all item loadings are over 0.6, whereas for AVE, which reflects the overall variance in the indicators accounted for by the latent construct, all exceeded the recommended value of 0.5 (Hair et al., 2013). In composite reliability (CR), only three constructs, ATT, PCB and SN are over the recommended value of 0.7.

Finally, the researcher tested the HTMT ratio to ensure discriminant validity, an alternative approach based on the multitrait-multimethod matrix, to assess discriminant validity^80^. For all constructs, as shown in Table 3, HTMT obtain values lower than 0.85, showing discriminant validity is satisfactory, and it poses a lesser threat to this study^81^.

**Table 3:** Heterotrait-monotrait (HTMT).

Construct	AM	CM	GM	ATT	PCB	INT	SN
Autonomous motivation							
Controlled motivation	0.501						
Gamification	0.626	0.35					
Attitude	0.835	0.603	0.707				
Perceived behavioral control	0.489	0.548	0.491	0.568			
Recycling intention	0.768	0.424	0.635	0.774	0.467		
Subjective norm	0.493	0.557	0.511	0.63	0.506	0.45	

Shaded boxes are the standard reporting format for HTMT procedure.

Source: Adapted from Henseler^80^, Hair et al.^77^ and data research.

### Structural model assessment

**Figure 3:**
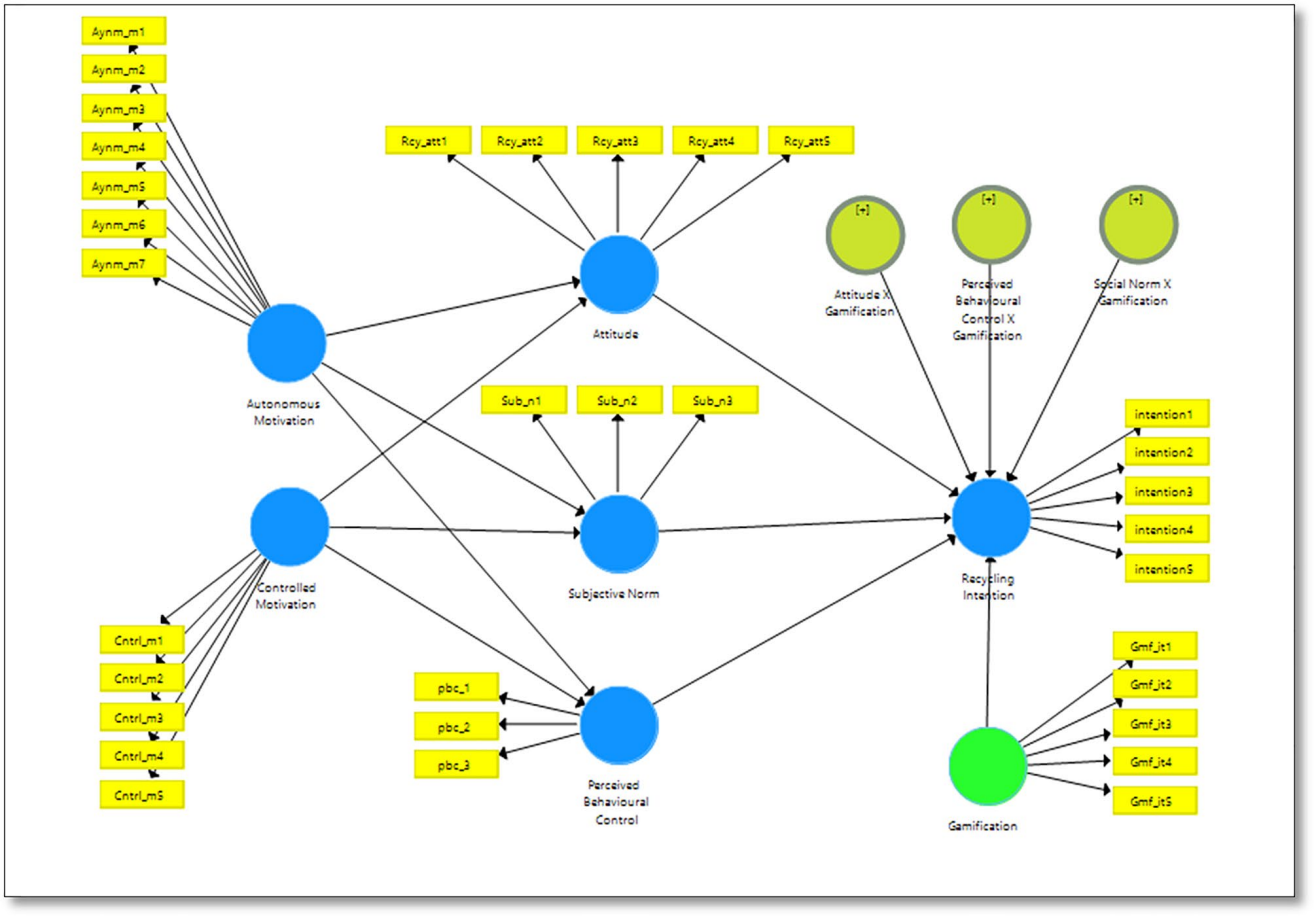
Research structural model from smartPLS.

As for the second stage, the researcher deemed 500 bootstrap samples adequate as it found support from a study by Deng et al.^82^, who found that the number of bootstrap replicates, ranging from 500 to 2000, had little effect on either bootstrap standard error or confidence interval to examine the significance of path coefficients. Figure 3 shows the structural model of the study from smartPLS.

Table 4 shows the hypothesis testing of the research. Controlled motivation is found to be a significant predictor of the relationship with attitude of youth’s recycling attitude, H1a (β = 0.324; p < 0.01), perceived behavioral control H1b (β = 0.352; p < 0.05), subjective norm H1c (β = 0.333; p < 0.01). Autonomous motivation is a significant predictor of attitude H2a (β = 0.597; p < 0.01) and subjective norm H2c (β = 0.288; p < 0.01). However, perceived behavioral control H2b is not supported (β = 0.274; p < 0.05).

**Table 4:** Structural estimates (hypothesis testing).

Hypotheses	Relationship (std beta)	Std error	T statistics	Decision	f^2^	Q^2^
H1a: Controlled motivation—> attitude	0.324	0.075	4.294**	Supported	0.220	0.070
H1b: Controlled motivation—> perceived behavioral control	0.352	0.131	2.578*	Supported	0.080	0.038
H1c: Controlled motivation—> Subjective Norm	0.333	0.145	2.291*	Supported	0.129	0.062
H2a: Autonomous motivation—> attitude	0.597	0.093	6.419**	Supported	0.819	0.300
H2b: Autonomous motivation—> perceived behavioral control	0.274	0.156	1.689*	Not supported	0.225	0.036
H2c: Autonomous motivation—> subjective norm	0.288	0.145	1.94*	Supported	0.094	0.042
H3a: attitude—> recycling intention	0.538	0.171	3.386**	Supported	0.391	0.305
H3b: Perceived behavioral control—> recycling intention	0.147	0.132	1.002	Not supported	0.03	0.002
H3c: Subjective norm- > recycling intention	− 0.007	0.149	0.088	Not supported	0	0.119
H4a: Attitude × gamification—> recycling intention	0.518	0.156	3.331**	Supported	0.262	0
H4b: Perceived behavioral control × gamification- > recycling intention	0.069	0.122	0.493	Not supported	0.006	0
H4c: Subjective norm × gamification—> recycling intention	0.023	0.148	0.064	Not supported	0	0
H5: Gamification—> recycling intention	0.225	0.146	1.578	Not supported	0.059	0.466

Critical t-values **2.58 (p < 0.01), *p < (0.05).

For the relationship between psychological determinant and recycling intention, it was found to be a significant predictor of attitude of youth’s recycling attitude, H2a (β = 0.597; p < 0.01), is subjective norm H2c (β = 0.343; p < 0.01), However, perceived behavioral control H2b is not supported (β = 0.274; p < 0.05).

For gamification as a moderator significant predictor for the relationship of attitude and recycling intention, H4a (β = 0.518; p < 0.01). Whereas, perceived behavioral control and recycling intention, H4b and subjective norm and recycling intention, H4c are not supported, (β = 0.069; p < 0.05) and (β = 0.023; p < 0.05). Lastly, the direct relationship between gamification and recycling intention, H5 is not supported (β = 0.225; p < 0.05).

### Assessment of the significance and relevance of structural model relationships

Coefficient of determination (R^2^), and effect size (f^2^). Besides R^2^ and f^2^, predictive relevance Q^2^ also measured for the inner model assessment for the research.

### R^2^ assessment

The coefficient of determination (R^2^), which measures the amount of explained variation of each endogenous latent variable, is the key criterion for inner model evaluation^83^. According to Cohen^84^, The R^2^ values of all constructs are higher than the 0.26, indicating the high effect size, whereas 0.13 to 0.25 indicate medium. It would indicate that this is a substantial model.

As shown in Table 5, R square adjusted explains 63.9% variance in attitude (R^2^ = 0.639), which is high effect size, whereas explains 27.2% variance in Perceived behavior control (R^2^ = 0.272), explains 66.1% variance in Recycling Intention (R^2^ = 0.661), and explains 27.9% variance in Subjective Norm (R^2^ = 0.278), which are medium effect size.

**Table 5:** R^2^ for the endogenous variable.

	R square
Attitude	0.639
Perceived behavioral control	0.272
Recycling intention	0.661
Subjective norm	0.278

### Effect size (f^2^) and predictive relevance (Q^2^) assessment

The following is to assess effect size (f^2^), it measures the magnitude of the effects and used broadly in behavioral sciences^85^. The effect size (f^2^) assesses a predictor’s contribution to a dependent variable. To measure the effect size, Cohen’s (1988) were used as guidelines, which are 0.02 for small effects, 0.15 for medium effects, and 0.35 for large effects. Table 6 shows that all relationships had a medium effect. In addition to the size of R^2^ and f^2^, the predictive sample reuse technique (Q^2^) can also effectively show predictive relevance^86^.

**Table 6:** R^2^ and Q^2^ values.

Endogenous constructs	R^2^	Q^2^
Autonomous motivation	0.434	0.185
Controlled motivation	0.119	0.023
Attitude	0.789	0.385
Perceived behavioral control	0.338	0.148
Recycling intention	0.720	0.319
Subjective norm	0.341	0.164

Source: Adapted from Hair et al.^87^ and data research.

Predictive relevance (Q^2^) is a criterion of predictive accuracy and evaluated through blindfolding, that resampling and omit data on endogenous variable and estimate for a complex model^80^. As shown in Table 6 summarizes the value of Q^2^ for Autonomous motivation (Q^2^ = 0.185), Controlled motivation (Q^2^ = 0.023), attitude (Q^2^ = 0.385), perceived behavioral control (Q^2^ = 0.148), recycling intention (Q^2^ = 0.319), subjective norm (Q^2^ = 0.164). All Q^2^ values are greater than 0 means that the model has predictive relevance for the constructs. (Hair et al., 2017).

### Verified conceptual model of motivation and recycling intention on gamified learning

**Figure 4:**
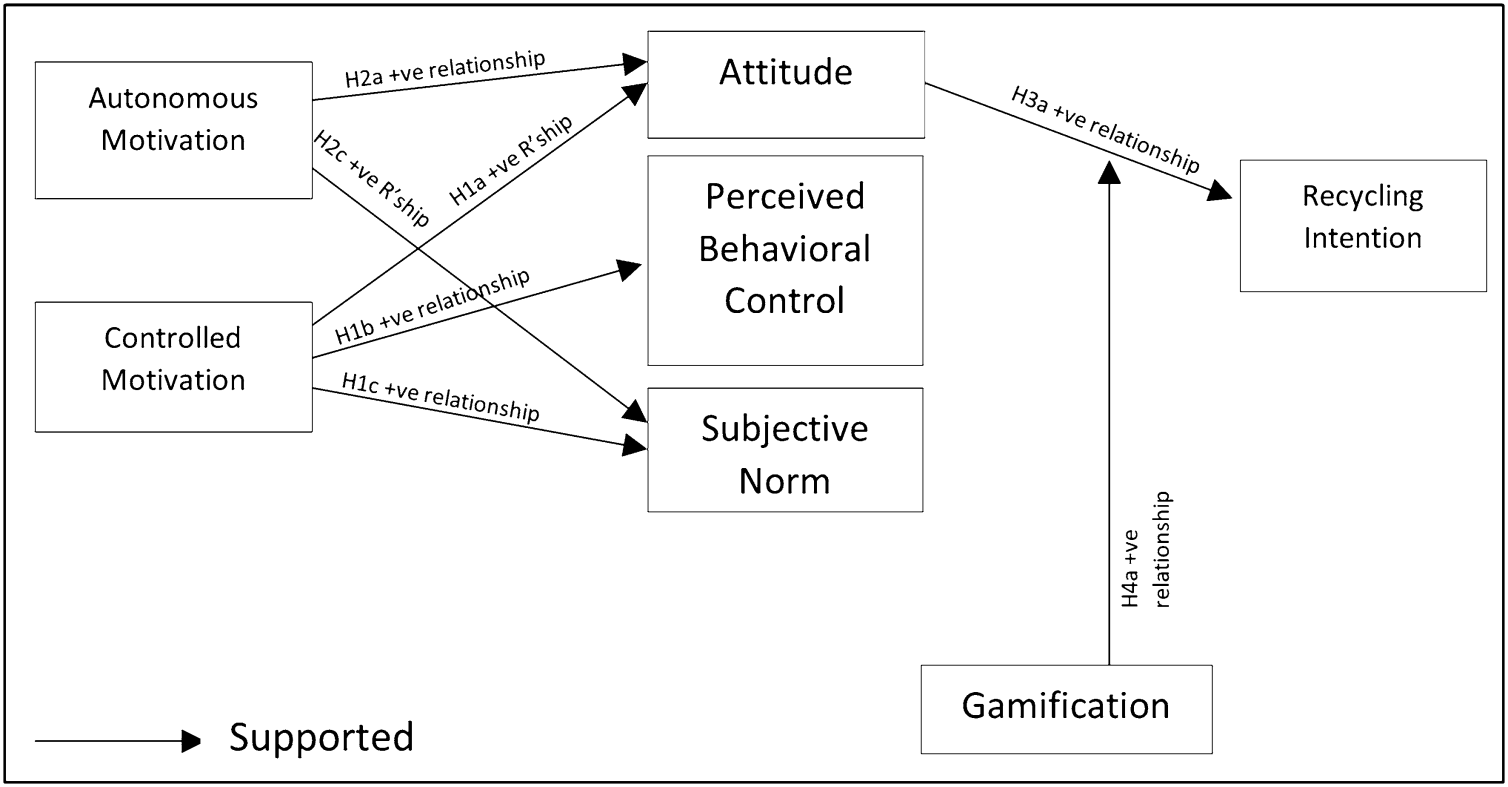
Verified conceptual model of motivation and recycling intention on gamified learning.

With the above results from inner and outer model measurement, as shown in Fig. 4, controlled motivation support all relationships of the psychosocial determinants, and autonomous motivation only support the relationship with attitude and subjective norm.

CM and AM are positively influenced attitude that leads to recycling intention. In contrast, perceived behavioral control and subjective norm do not support the relationship with recycling intention. It has clearly shown that in an EGL activity, both CM and AM can affect the youth's recycling attitude and positively lead to recycling intention. However, Gamified learning only moderates the relationship between attitude and recycling intention.

## Discussion

Recycling intention for youth is considered an effective way towards a sustainable environment. Motivation to recycle is part of the drive for a better attitude. Recycling campaigns, activities and actions in Malaysia is increasing, but the awareness still needs to be spread to more people, especially youth, to reach a higher rate like nearby Asian countries, Singapore, Korea and Hong Kong. This study explores the gamified recycling activity towards recycling intention for youth to clarify the motivation of gamified learning towards recycling intention. The hypotheses are not fully supported and accepted, but H1 to H3 answer RQ 1 on how AM, CM and psychosocial determinants enhance recycling intention. Following the two hypotheses H4 and H5, both answers RQ 2 that the difference of recycling intention is associated with or without gamified learning.

For H1, controlled motivation (CM) has a positive association with attitude, perceived behavioral control, and social norm of recycling, which means that Controlled motivation supports three main psychosocial determinants of TPB in recycling. Controlled motivation is the external regulation, and the youth may expect to receive a reward or perceived approval from their friends and judges from the leaderboard that works positively in the gamified recycling learning activity.

For H2, Autonomous motivation has a positive association with attitude and social norm but not perceived behavioral control. This show that the youth have a positive mindset towards the gamified activity, and social pressure also are positive that caused the positive effect of social norm in recycling. But not supported in PBC, and through the result, it is found that how easy or difficult the activity perceived by the participants are not related to their own will to work on the recycling activity.

For H3, only attitude from the three psychosocial determinants is associated positively with youth’s recycling intention. The positive relationship has proven that the recycling intention is carried out based on the favoritism of the youth but not the other two psychosocial determinants: the influence from friends and the perception of the difficulty of recycling intention.

H4, gamification has not functioned as a moderator in perceived behavioral control and social norm but only supported the attitude in recycling intention. From the result, gamification moderated the youth’s favoritism towards the recycling intention, and from this relationship, it can be perceived that gamified learning has given the youths a positive mindset towards recycling intention.

Lastly, H5, gamification is not positively associated with recycling intention. It shows that if the gamified environment does not incorporate attitude, it will not work independently to achieve recycling intention.

Overall, Controlled motivation is more effective than autonomous motivation towards perceived behavioral control, where autonomous motivation would not lead to the youth’s perceived difficulty in recycling intention. This study shows that not all relationships are positive in a gamified learning environment, and it gives a good view of the weaknesses and strengths of gamified learning with the guideline of SDT and TPB.

## Limitation

This study only used part of the SDT and TPB where SDT only on autonomous and controlled motivation, whereas SDT only up to behavioral intention, where the end goal of TPB is often at behavior. The research data collected by the online questionnaire is non-random sampling data, and the number of the responses is relatively small, only 60. All the respondents are volunteers. The theories used from SDT and TPB are used partially to scope the study effectively. Additionally, the participants, which is less but adequate for smartPLS can address the hypotheses and answer the research questions.

## Implications

Multiple implications may be applied practically based upon the result of the research. First, establishment and improvement of existing gamified model using SDT and TPB as the theory of research in learning.

The study model shown in Fig. 2 displays relationships of the constructs from TPB and SDT in a gamified environment. It will contribute the knowledge to learning and social sciences researchers to study how gamified learning motivates the participation of recycling among the youth by further improving the TPB and SDT. A positive relationship from Autonomous motivation to attitude and led to intention have shown that both theories of TPB and SDT are interlinkable through the autonomous motivation from SDT with the attitude from TPB to achieve a positive recycling intention, gamification also plays its role as a moderator to strengthen the relationship between attitude and recycling intention. Therefore, with proper implementation or treatment of the gamification elements could also make learning activities fun, and this study will contribute to the knowledge of motivation and intention on the context of fun learning in recycling among youth especially with the assistance of the technological aspects^7^.

Second, educating the youth regarding recycling and its benefits from gamified activity (Edcraft Gamified Learning). The knowledge and understanding regarding recycling may be enhanced through more mechanisms of gamification. Although after experiencing the gamified activity, gamification could not directly affect PBC and SN towards recycling intention, it has a significant effect on the attitude towards the recycling of the youth. Hence, gamified learning activity of this study gives a positive thought for the youth in recycling intention. This is beneficial to the environment due to the increase of household waste varieties, from plastic to e-waste, it is important that more products can be repurposed or recycled to cope with the sustainable cities and communities (SDG 11) and with lesser unmanaged waste, ecosystems are less harmed that will lead to the climate action (SDG 13).

Gamification has proven its role in positively influencing the favoritism of youth towards recycling intention. Findings from this study has led to a new path on the practicality of gamification of recycling activity in mobile platforms to access more people in the future, e.g., WeChat platform to conduct a broader range of users around a variety of user range. Moreover, improper placement of recycling in ineffective digital platforms will negatively impact attitudes towards the environment^88^. By having a good platform such as a mobile app or easily accessible information technology tools for recycling can apply to Malaysia or other countries to collect a broader range of data among youth.

## Conclusion

This research focuses on gamified learning on some of the mechanisms that evoked controlled and autonomous motivation for their attitude and recycling intention, especially youths who play an essential role in sustainable cities and sustainable development goals. However, the study design does not include other video game mechanisms such as progress bar, experience pointer, or bosses. Therefore, the relationship of gamification with motivation and intention of recycling still can go beyond its current scope. More constructs like motivation, the non-motivated action from SDT and behavior from TPB can be further extended in a future study.

## Data Availability

All data analyzed or generated are available in the paper.
